# Deep learning-based quantitative analysis of glomerular morphology in IgA nephropathy whole slide images and its prognostic implications

**DOI:** 10.1038/s41598-025-09031-w

**Published:** 2025-07-02

**Authors:** Seung Yeon Cho, Yisak Kim, Sehoon Park, Jin Ho Paik, Ho Jun Chin, Jeong Hwan Park, Jung Pyo Lee, Yong-Jin Kim, Sun-Hee Park, Ho-chang Lee, Hyunjeong Cho, Beom Jin Lim, Hyung Woo Kim, Seung Hyeok Han, Heounjeong Go, Chung Hee Baek, Hajeong Lee, Kyung Chul Moon, Young-Gon Kim

**Affiliations:** 1 Interdisciplinary Program in Bioengineering, Graduate School, Seoul National University, Seoul, Republic of Korea; 2 Integrated Major in Innovative Medical Science, Seoul National University College of Medicine, Seoul, Republic of Korea; 3 Department of Internal Medicine, Seoul National University Hospital, Seoul, Republic of Korea; 4 Department of Pathology, Seoul National University Bundang Hospital, Seoul National University College of Medicine, Seongnam, Republic of Korea; 5 Department of Internal Medicine, Seoul National University College of Medicine, Seoul, Republic of Korea; 6 Department of Internal Medicine, Seoul National University Bundang Hospital, Seongnam, Republic of Korea; 7 Department of Pathology, Seoul Metropolitan Government Seoul National University Boramae Medical Center, Seoul, Republic of Korea; 8 Department of Internal Medicine, Seoul National University Boramae Medical Center, Seoul, Republic of Korea; 9 Department of Pathology, School of Medicine, Kyungpook National University Hospital, Kyungpook National University, Daegu, Republic of Korea; 10 Department of Internal Medicine, Kyungpook National University School of Medicine, Kyungpook National University Hospital, Daegu, Republic of Korea; 11 Department of Pathology, Chungbuk National University College of Medicine, Cheongju, Republic of Korea; 12 Department of Internal Medicine, Chungbuk National University Hospital, Chungbuk National University College of Medicine, Cheongju, Republic of Korea; 13 Department of Pathology, Yonsei University College of Medicine, Seoul, Republic of Korea; 14 Department of Internal Medicine, Institute of Kidney Disease Research, Yonsei University College of Medicine, Seoul, Republic of Korea; 15 Department of Pathology, Asan Medical Center, University of Ulsan College of Medicine, Seoul, Republic of Korea; 16 Department of Internal Medicine, Asan Medical Center, University of Ulsan College of Medicine, Seoul, Republic of Korea; 17 Department of Pathology, Seoul National University College of Medicine, Seoul, Republic of Korea; 18 Department of Pathology, Seoul National University Hospital, 101 Daehak-ro, Jongno-gu, Seoul, 03080 Republic of Korea; 19 Department of Transdisciplinary Medicine, Seoul National University Hospital, 101 Daehak-ro, Jongno-gu, Seoul, 03080 Republic of Korea; 20 Department of Medicine, Seoul National University College of Medicine, Seoul, Republic of Korea

**Keywords:** Diagnostic markers, Biomedical engineering, Kidney diseases, Medical imaging, Prognosis, Outcomes research

## Abstract

Kidney pathology of immunoglobulin A nephropathy (IgAN), which is the key finding of both diagnosis and risk stratification, involves labor-intensive manual interpretation as well as unavoidable interpreter-dependent variabilities. We propose artificial intelligence-based frameworks for quantitatively analyzing glomerular histologic features that can predict kidney progression in IgAN. A deep learning model, based on DeepLabV3Plus and EfficientNet-B3, was developed for segmenting glomeruli and quantifying the morphological features by using digitized whole slide images from seven tertiary hospitals. Subsequently, it was used for machine learning-based risk prediction of IgAN progression. Its predictability was compared with the conventional clinicopathologic feature-based model to demonstrate its comparable performance. In total, 1,241 whole slide images were obtained. The weighted averages of average precision and dice similarity coefficient were 0.795 and 0.721 in internal validation and 0.818 and 0.743 in external validation, respectively. Interestingly, image features-only-based kidney outcome prediction models showed similar predictability compared with clinical features-only-based models. In addition, incorporating an image-based deep learning model into the clinical features-based models enhanced predictabilities, although insignificant. These results show that quantitative glomerular histologic features are comparable to clinical data, suggesting that they may offer additional prognostic insights not covered by Oxford classification or other clinical parameters.

**Supplementary Information:**

The online version contains supplementary material available at 10.1038/s41598-025-09031-w.

## Introduction

Immunoglobulin A nephropathy (IgAN) is one of the most prevalent glomerular diseases worldwide, contributing substantially to the progression of kidney failure^1,2^. It is characterized by the abnormal deposition of immunoglobulin A, leading to subsequent inflammation of glomeruli^3^. Accurate and timely diagnosis of IgAN and its risk assessment are necessary for guiding appropriate clinical management, as early intervention can potentially slow or prevent disease progression. Kidney biopsy is an essential routine clinical procedure for patients with glomerular diseases, providing diagnostic and prognostic information to guide clinical decision-making^3,4^. Although it remains the gold standard for the diagnosis and risk assessment of IgAN, the interpretation of kidney biopsy slides is complex and labor-intensive, requiring the expertise of pathologists.

Grading systems and prognostic tools have been developed to better characterize IgAN and predict progression outcomes^5,6^. Particularly, the Oxford classification (the MEST-C score) of IgAN has been reported as a highly successful prognostic classification system in nephropathology, standardizing the biopsy reports and improving reliability^5,6^. However, Oxford classification requires the manual examination of the biopsy slides and involves the examiner’s visual estimation. Such inherent subjectivity in histopathological assessments may lead to intra- and inter-observer variations, underscoring the need for complementary approaches^7–9^. Recently, the International IgAN Prediction Tool (IIgAN-PT), a risk prediction model, was introduced, leveraging clinical and histological data to quantify the risk of kidney disease progression at diagnosis^10^. Although it demonstrated significant advancement in the prognostic modeling of IgAN, it incorporates Oxford classification, and its utility may be limited by the availability and completeness of the data required^10^.

In recent years, the integration of digital pathology and artificial intelligence techniques has opened new avenues for enhancing the reliability and efficiency of kidney biopsy interpretation, gaining increasing attention from the medical community^11–14^. Specifically, deep learning-based glomerulus segmentation has been employed to analyze glomerulosclerosis in whole slide images (WSIs) of Periodic acid-Schiff (PAS) biopsy specimens^15,16^. Acknowledging the significance of quantifying glomeruli for the histopathologic assessment of kidney tissue, studies have developed an ensemble of deep learning models to further improve the performance of glomerulus segmentation^17^. In addition, some machine learning methods have been applied to identify critical clinical and histopathological predictors of disease progression in minimal change disease or focal segmental glomerulosclerosis^18^. Considering these advancements, encompassing an artificial intelligence approach to analyze various characteristics of glomerular lesions from the biopsy images of IgAN patients may offer additional valuable information to clinicians.

This study aimed to develop and validate a AI-based framework to automatically quantify and analyze glomerular histologic features for prognostic classification of IgAN using a multi-center cohort. Various types of glomerular lesions were segmented and classified by deep learning models, and the morphological features of the glomeruli were extracted to predict the progression of IgAN. In addition, image-based and clinical data-based prognostic models were compared to evaluate whether glomerular histologic features provide prognostic value comparable to that of clinical parameters. This study is novel in that it integrates lesion-specific glomerular segmentation with quantitative morphometric analysis across a large, multi-center PAS-stained biopsy cohort, and demonstrates that even basic image-derived features can achieve prognostic performance comparable to clinical models.

## Materials and methods

### Study subjects

This study was a retrospective, multi-center study considering biopsy-confirmed cases with primitive IgAN diagnosis. The cases were obtained from seven different medical institutions in South Korea—Seoul National University Hospital (SNUH), Seoul National University Bundang Hospital (SNUBH), Seoul Metropolitan Government Seoul National University Boramae Medical Center (SMG-SNU BMC), Kyungpook National University Hospital (KNUH), Chungbuk National University Hospital (CBNUH), Severance Hospital (SVH), and Asan Medical Center (AMC). All patients included in this study had digital biopsy images available; corresponding demographic, clinical, and pathological characteristics were also collected (Table 1). For the clinical variables, estimated glomerular filtration rate (eGFR), serum creatinine levels, and urine protein-to-creatinine ratio (UPCR) were included, collectively describing the kidney function. In addition, only the scores available through pathology reports were included for the pathological variables. Exclusion criteria were any biopsy slides of inferior quality, as the primary input for the glomerulus segmentation model was the biopsy images; cases with less than six months of follow-up were further excluded during the development of the prognostic classification model.

**Table 1:** Clinical and pathologic characteristics of the study population.

	Internal set			External set
	Train	Development	Validation	Validation
Sex				
Male	278 (54.8%)	55 (50.5%)	47 (43.1%)	255 (49.4%)
Age [years]	33.93 (18.96)	35.92 (16.21)	38.75 (18.10)	41.62 (14.93)
BMI [kg/m^2^]	22.40 (3.94)	23.51 (4.35)	23.10 (3.97)	24.55 (4.25)
eGFR [mL/min/1.73m^2^]	93.87 (46.15)	87.49 (38.91)	90.49 (43.84)	80.89 (34.70)
UPCR [g/g or g/24 h]	1.93 (2.59)	1.43 (1.36)	1.91 (1.93)	1.74 (2.11)
Creatinine [mg/dL]	1.26 (1.30)	1.14 (0.59)	1.06 (0.56)	1.18 (0.99)
Diabetes Mellitus	21 (4.16%)	10 (9.17%)	6 (5.50%)	32 (6.20%)
Hypertension	180 (35.64%)	40 (36.70%)	37 (33.95%)	187 (36.24%)
Systolic BP [mmHg]	118.49 (13.96)	121.24 (14.03)	118.82 (14.90)	126.73 (17.45)
Diastolic BP [mmHg]	72.85 (11.29)	75.48 (11.18)	73.79 (11.73)	78.02 (11.88)
ISD	54 (10.65%)	7 (6.42%)	11 (10.09%)	16 (3.10%)
RASB	251 (49.51%)	58 (53.21%)	54 (49.54%)	217 (42.05%)
Hemoglobin	12.94 (1.86)	13.10 (1.62)	12.83 (1.87)	13.05 (1.91)
MAP [mmHg]	88.06 (11.41)	90.73 (11.13)	88.80 (12.22)	94.26 (12.68)
Kidney outcome				
0	413 (81.5%)	96 (88.1%)	95 (87.2%)	437 (90.1%)
1	94 (18.5%)	13 (11.9%)	14 (12.8%)	48 (9.9%)
SMK Lee				
1	22 (5.4%)	5 (4.6%)	3 (2.8%)	1 (0.9%)
2	131 (31.9%)	51 (47.2%)	61 (56.5%)	11 (10.4%)
3	181 (44.0%)	40 (37.0%)	33 (30.6%)	74 (70.0%)
4	60 (15.6%)	6 (5.6%)	10 (9.3%)	18 (17.0%)
5	17 (4.1%)	6 (5.6%)	1 (0.9%)	2 (1.9%)
Haas				
1	22 (5.4%)	4 (3.7%)	2 (1.9%)	66 (17.8%)
2	26 (6.3%)	8 (7.4%)	8 (7.4%)	41 (11.1%)
3	106 (25.8%)	43 (39.8%)	51 (47.2%)	108 (29.1%)
4	207 (50.4%)	43 (39.8%)	35 (32.4%)	101 (27.2%)
5	50 (12.2%)	10 (0.9%)	12 (11.1%)	55 (14.8%)
Oxford				
M1	122 (43.1%)	36 (33.0%)	33 (30.3%)	196 (46.5%)
E1	56 (19.8%)	26 (23.9%)	42 (38.5%)	90 (21.4%)
S1	171 (60.4%)	71 (65.1%)	77 (70.6%)	306 (72.5%)
T1	46 (16.2%)	20 (18.3%)	17 (15.6%)	118 (28.0%)
T2	22 (7.8%)	5 (4.6%)	5 (4.6%)	22 (5.2%)
C1	69 (13.6%)	18 (16.5%)	24 (22.0%)	96 (22.0%)
C2	6 (1.2%)	0	0	5 (1.2%)

Continuous variables are presented as mean (standard deviation) and categorical variables as number (percentage).

*BMI* body mass index, *eGFR* estimated glomerular filtration rate, *UPCR* urine protein-to-creatinine ratio, *BP* blood pressure, *ISD* immunosuppressive drug, *RASB* renin–angiotensin–aldosterone blockades, *MAP* mean arterial pressure.

### Study outcome

The primary study outcome included a reduction in eGFR to below 50% of the value at biopsy or the occurrence of end-stage kidney disease (eGFR < 15 mL/min/1.73 m^2^ or kidney replacement therapy). The cohort was censored at the occurrence of the outcome event or the point of follow-up loss.

### Data description

**Fig. 1:**
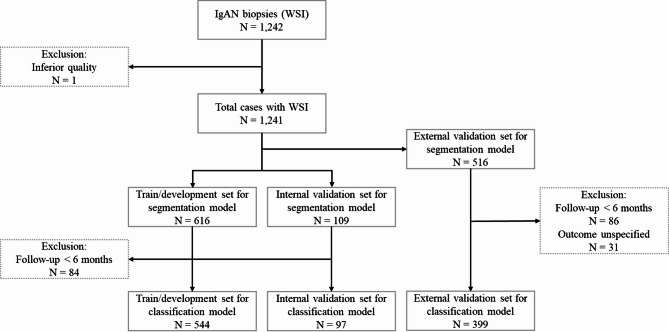
Flowchart of case selection, exclusion, and split in the training/development and validation sets. Flowchart of case selection and dataset split, summarizing the inclusion and exclusion criteria and illustrates how the dataset was divided into training, development, and validation sets.

The slides from SNUH, SNUBH, BRMH, and parts of KNUH were acquired in ScanScope Virtual Slide (SVS) format, and those from AMC were acquired in TIF format. The rest of the biopsy images were provided as microscopic glass slides and scanned using a digital microscopy scanner (Aperio AT2; Leica Biosystems, Wetzlar, Germany) in SVS format at SNUH; these digitized slides are referred to as WSIs. For each WSI obtained, nephropathologists annotated a single tissue core and glomeruli. The manually scanned and TIF slides were not annotated. Each annotated glomerulus was labeled as one of the five classes that describe the lesion types: no lesion, global sclerosis, segmental sclerosis, crescent, or ischemic change (Table 2). The annotations underwent two-stage validation by expert nephropathologists, where one group labeled the glomeruli, and another group validated the labels. As WSIs from SNUH reflected the most substantial data collection, they were split into training, development, and validation sets; WSIs from the remaining six institutions were used for external validation. The WSIs containing minor class glomeruli were prioritized to be split to establish balanced training and validation sets. This ensured that minor class glomeruli, characterized by a lower prevalence, were distributed proportionally across both sets. All WSIs used in this study were PAS-stained specimens. A detailed overview of case inclusion, exclusion criteria, and how the dataset was split into training, development, and validation sets is provided in Fig. 1. This diagram summarizes the flow of data collection and preparation used for model development and evaluation.

**Table 2:** Number of annotated glomeruli in the internal and external cohort.

	Internal set			External set
	Train	Development	Validation	Validation
Cases	**507**	**109**	**109**	**516**
NL	6,504	1,439	1,579	2,386
GS	1,853	461	410	761
SS	539	98	126	231
CR	171	50	31	129
ISC	142	42	31	93
Total glomeruli	9,209	2,090	2,177	3,600

*NL* no lesion, *GS* global sclerosis, *SS* segmental sclerosis, *CR* crescent, *ISC* ischemic change.

### Model development

#### Data pre-processing

A patch-based approach using a sliding window of patch size 512 × 512 was used for training the segmentation model. The sliding window size was set as 50% overlap for each patch to ensure that all biopsy regions were adequately covered; the image patches that did not contain any pixels of glomerulus were discarded. All patches were extracted at 10$\:\times\:$ magnification of the slide level. Next, sampling methods were applied to mitigate the impact of class imbalances, as the number of no-lesioned glomeruli largely outnumber the lesioned glomeruli. In this study, combined sampling was used to overcome class imbalance leading to overfitting, where patches with no-lesioned glomeruli were under-sampled, and patches containing the rest of the classes were over-sampled. Finally, various data augmentations were performed on each input image using the Albumentation library before being input to the model^19^.

#### Segmentation model

The deep learning model aimed to segment the glomeruli in WSIs and classify the lesion type. Instead of concatenating segmentation and classification models, a multi-class segmentation model was designed to segment and classify the glomeruli simultaneously. Therefore, the input data comprised an RGB image of a tissue patch and a 5-channel mask image, with each channel representing a mask for one of the five glomerulus classes. The model was trained using DeepLabV3Plus with EfficientNet-B3 as an ImageNet-pretrained backbone encoder^20,21^. The Adam optimizer was applied, and the Dice loss was selected for the loss function. The initial learning rate was 1$\:{e}^{-4}\:$ with a step decay factor of 0.5 after every 10 epochs of no improvement in loss; the training was stopped after another 10 epochs with no improvement in loss. During validation, patch-wise Test Time Augmentation^22^, including flip, rotation, and multiply, was applied to further improve the segmentation performance. The patch-level inference results were aggregated to return a final slide-level inference.

#### Data post-processing

After the glomerulus segmentation, a post-processing step was conducted to address the issue of connected over-segments within the segmented regions, where two or more glomeruli located closely are segmented to connect the boundaries of the masks. First, the distance transform was applied to generate a distance map of the segmented glomeruli; this encoded the proximity of each pixel to the nearest background pixel. Subsequently, the watershed algorithm used the markers derived from the local minima in the distance map to expand the regions gradually. This process effectively separated the connected over-segments into distinct, non-overlapping glomerulus instances.

#### Morphological feature extraction

The predicted mask images of glomeruli with various types of lesions were used to analyze the morphological characteristics of the predicted glomeruli. Basic computer vision techniques were used to compute the area (number of pixels) of glomeruli, number of glomeruli, length of major and minor axes, solidness, compactness, eccentricity, and roundness (Supplementary Table S2). Each feature was extracted separately for each glomerular lesion class and averaged across glomeruli to represent the slide-level feature. For the area and number of glomeruli, the ratio of the value of each class relative to the total was calculated. The intention of using these features was to evaluate whether basic, simple, and explainable features that are directly measurable from glomerular morphology could provide meaningful prognostic information at the slide level.

#### Prognostic classification model

**Fig. 2:**
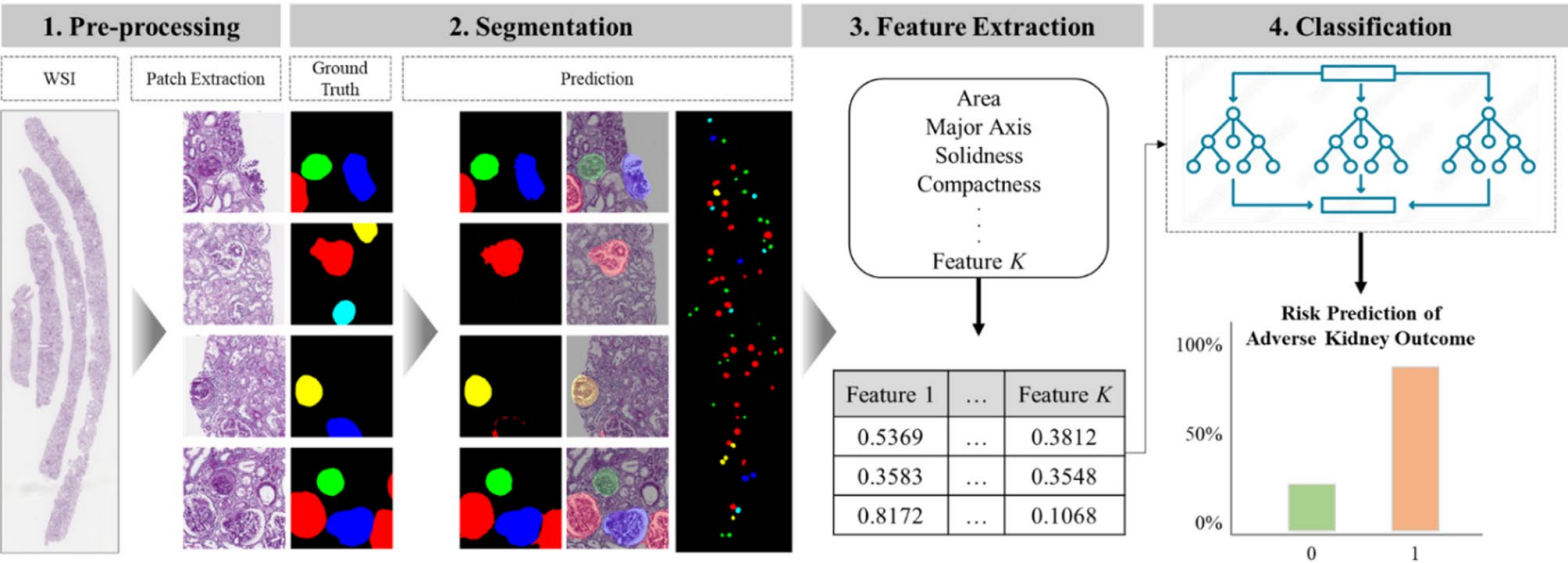
Schematic workflow of the proposed framework. This diagram illustrates the end-to-end process of the study, including patch extraction of input slides, glomerular segmentation by lesion type, morphological feature extraction, and machine learning-based prognosis prediction. Eash lesion type is color-coded: red (no lesion), green (global sclerosis), blue (segmental sclerosis), yellow (crescent), cyan (ischemic change).

As the final stage of the proposed framework, classification models were trained using the previously extracted image-based features to predict kidney disease prognosis in IgAN. The comparability of the image-based prognostic model was compared against two other models trained using clinical information: one model was trained using the basic clinical data collected in the electronic medical record, and another was trained based on the variables used for IIgAN-PT. The complete list of input variables included for each model can be found in Supplementary Table S1. Additionally, the input features for the two clinical data-based models were combined with the image-based features for further assessment. Training was conducted using three classic machine learning algorithms, namely extreme gradient boosting (XGBoost), random forest, and logistic regression^23–25^. The Scikit-learn modules were used for the machine learning library^26^. A step-by-step schematic of the full workflow, including input WSIs, glomerular segmentation, feature extraction, and prognostic classification, is illustrated in Fig. 2.

### Statistical analysis

The performance of the segmentation model was evaluated in terms of average precision (AP) and dice similarity coefficient (DSC) scores, each with 95% confidence intervals (CI). For AP, a glomerulus prediction was considered a true positive when the predicted region overlapped with at least 50% of a ground-truth region. Both metrics were evaluated for each class of glomeruli, and the weighted average scores were computed for internal and external validation sets to compare the overall performance. The performances of the classification models were assessed through a receiver operating characteristic (ROC) analysis and evaluated using the area under the ROC curve (AUC). The AUC values of the classification models were compared using Delong’s test^27^. All statistical analyses were performed using the Python environment (v.3.8.0). The level of statistical significance was set at *p* < 0.05.

### Ethical statement

The study protocols were approved by the Institutional Review Board Committees of SNUH/SNUBH/SMG-SNU BMC (IRB number: H-2103-091-1205), KNUH (IRB number: 2021-04-036), CBNUH (IRB number: 2021-09-004), SVH (IRB number: 4-2021-0376), and AMC (IRB number: 2021 − 1333), which waived the need for informed patient consent. The study adhered to the principles of the Declaration of Helsinki.

### Code availability

The code used for morphological feature extraction, prognostic model development, and evaluation is available via GitHub: https://github.com/younggon2/Research-Segmentation_Glomerulus.

## Results

### Study subjects

A total of 1,241 digitized kidney biopsies of patients diagnosed with IgAN were acquired from seven medical institutions in South Korea. Of these, 725 slides were from SNUH, which were further split into 507 and 109 slides for training and development sets, respectively, for training the segmentation model. The remaining 109 slides were used as the validation set. For external validation, 520 kidney biopsies were additionally obtained from six other institutions: 77 slides from SNUBH, 109 slides from KNUH, 20 slides from SMG-SNU BMC, 145 slides from CBNUH, 81 slides from SVH, and 88 slides from AMC. The slides from SNUBH, SMG-SNU BMC, and parts of KNUH had a resolution of 0.25 $\:\mu\:$m per pixel (MPP), and those from AMC had a resolution of 0.21 MPP. The rest, provided as microscopic glass slides, were scanned with a resolution of 0.25 MPP. One WSI was excluded before the pre-processing step owing to its inferior quality. At the prognostic classification stage, 84 and 86 cases from the internal and external sets, respectively, with follow-up durations of less than 6 months, were excluded. Additionally, 31 cases were excluded from the external set due to unspecified kidney outcomes. In the internal and external sets, cases with adverse kidney outcomes comprised less than 20% and 10%, respectively (Table 1), which poses a potential challenge in classification because of class imbalance^28^.

### Segmentation model

The performance of the segmentation model was evaluated in terms of AP and DSC scores (95% CIs). The AP scores were 0.834 (0.803–0.865), 0.785 (0.730–0.840), 0.505 (0.423–0.587), 0.446 (0.354–0.538), and 0.438 (0.350–0.526) for glomeruli with no lesion, global sclerosis, segmental sclerosis, crescent, and ischemic change, respectively. The corresponding DSC scores were 0.792 (0.761–0.823), 0.574 (0.514–0.634), 0.474 (0.398–0.550), 0.336 (0.246–0.426), and 0.342 (0.256–0.428). In external validation, the AP scores obtained were 0.854 (0.832–0.876), 0.795 (0.749–0.841), 0.617 (0.556–0.678), 0.472 (0.408–0.536), and 0.447 (0.379–0.515) for glomeruli with no lesion, global sclerosis, segmental sclerosis, crescent, and ischemic change, respectively, and corresponding DSC scores were 0.805 (0.787–0.823), 0.638 (0.588–0.688), 0.472 (0.411–0.533), 0.419 (0.355–0.483), and 0.360 (0.293–0.427). The weighted average of AP was 0.795 (0.735–0.854) and 0.818 (0.756–0.848) in internal and external validation, respectively, and the corresponding weighted average of DSC was 0.721 (0.660–0.779) and 0.743 (0.677–0.769). The glomerulus segmentation results are summarized in Table 3.

**Table 3:** Performances of the deep learning-based glomeruli multi-segmentation model.

	Internal validation		External validation	
	AP	DSC	AP	DSC
No lesion	*n* = 1,579		*n* = 2,386	
	0.834 (0.803–0.865)	0.792 (0.761–0.823)	0.854 (0.832–0.876)	0.805 (0.787–0.823)
Global sclerosis	*n* = 410		*n* = 761	
	0.785 (0.730–0.840)	0.574 (0.514–0.634)	0.795 (0.749–0.841)	0.638 (0.588–0.688)
Segmental sclerosis	*n* = 126		*n* = 231	
	0.505 (0.423–0.587)	0.474 (0.398–0.550)	0.617 (0.556–0.678)	0.472 (0.411–0.533)
Crescent	*n* = 31		*n* = 129	
	0.446 (0.354–0.538)	0.336 (0.246–0.426)	0.472 (0.408–0.536)	0.419 (0.355–0.483)
Ischemic change	*n* = 31		*n* = 93	
	0.438 (0.350–0.526)	0.342 (0.256–0.428)	0.447 (0.379, 0.515)	0.360 (0.293–0.427)
Weighted average	*n* = 2,177		*n* = 3,600	
	0.795 (0.735–0.854)	0.721 (0.660–0.779)	0.818 (0.756–0.848)	0.743 (0.677–0.769)

Values are presented as mean (range of 95% confidence interval); AP, average precision; DSC, dice similarity coefficient.

### Morphological feature extraction

The quantified values of the morphological features of glomeruli extracted from the segmented masks are summarized in Table 4. On average, glomeruli covered 9.6% of the entire tissue region in the WSI. Glomeruli with no lesion occupied the largest proportion among the different glomerular lesion types, with a mean area of 69.3%. This result aligns with the fact that the number of glomeruli with no lesion is the highest among all types. On average, the number of glomeruli with no lesion was 44.2% in each slide, and the number of global sclerotic glomeruli was 37.6%, the second most prevalent. By contrast, glomeruli with ischemic changes were the least common, with an average of only 4.4% per slide.

**Table 4:** Morphological features of glomeruli extracted from the total validation cohort.

	NL	GS	SS	CR	ISC	Total
Area [%]	69.3 (0.22)	16.0 (0.15)	7.8 (0.08)	3.9 (0.07)	3.0 (0.05)	9.6 (5.8)^a^
Count [%]	44.2 (0.24)	37.6 (0.23)	8.4 (0.08)	5.4 (0.08)	4.4 (0.06)	N/A
Major axis [$\:{\upmu\:}\text{m}]$	205.3 (33.82)	132.6 (44.20)	138.7 (90.43)	87.0 (87.57)	90.4 (83.13)	126.2 (83.28)^b^
Minor axis [$\:{\upmu\:}\text{m}$]	157.1 (25.71)	104.1 (34.26)	105.8 (72.81)	64.7 (68.04)	66.3 (63.65)	95.6 (64.71)
Compactness	1.250 (0.16)	1.178 (0.28)	1.012 (0.60)	0.813 (0.81)	0.850 (0.75)	1.003 (0.61)
Eccentricity	0.820 (0.10)	0.816 (0.20)	0.617 (0.37)	0.428 (0.41)	0.473 (0.41)	0.620 (0.37)
Solidity	0.965 (0.10)	0.944 (0.19)	0.743 (0.42)	0.527 (0.48)	0.580 (0.47)	0.742 (0.42)
Roundness	0.799 (0.10)	0.794 (0.18)	0.588 (0.34)	0.389 (0.37)	0.445 (0.38)	0.597 (0.35)

Values are presented as mean (standard deviation).

*NL* no lesion, *GS* global sclerosis, *SS* segmental sclerosis, *CR* crescent, *ISC* ischemic change.

^a^Average total area of glomeruli over total area of entire tissues, ^b^For the shape features, mean (standard deviation) values of all types of glomeruli are calculated.

### Prognostic classification model

**Fig. 3:**
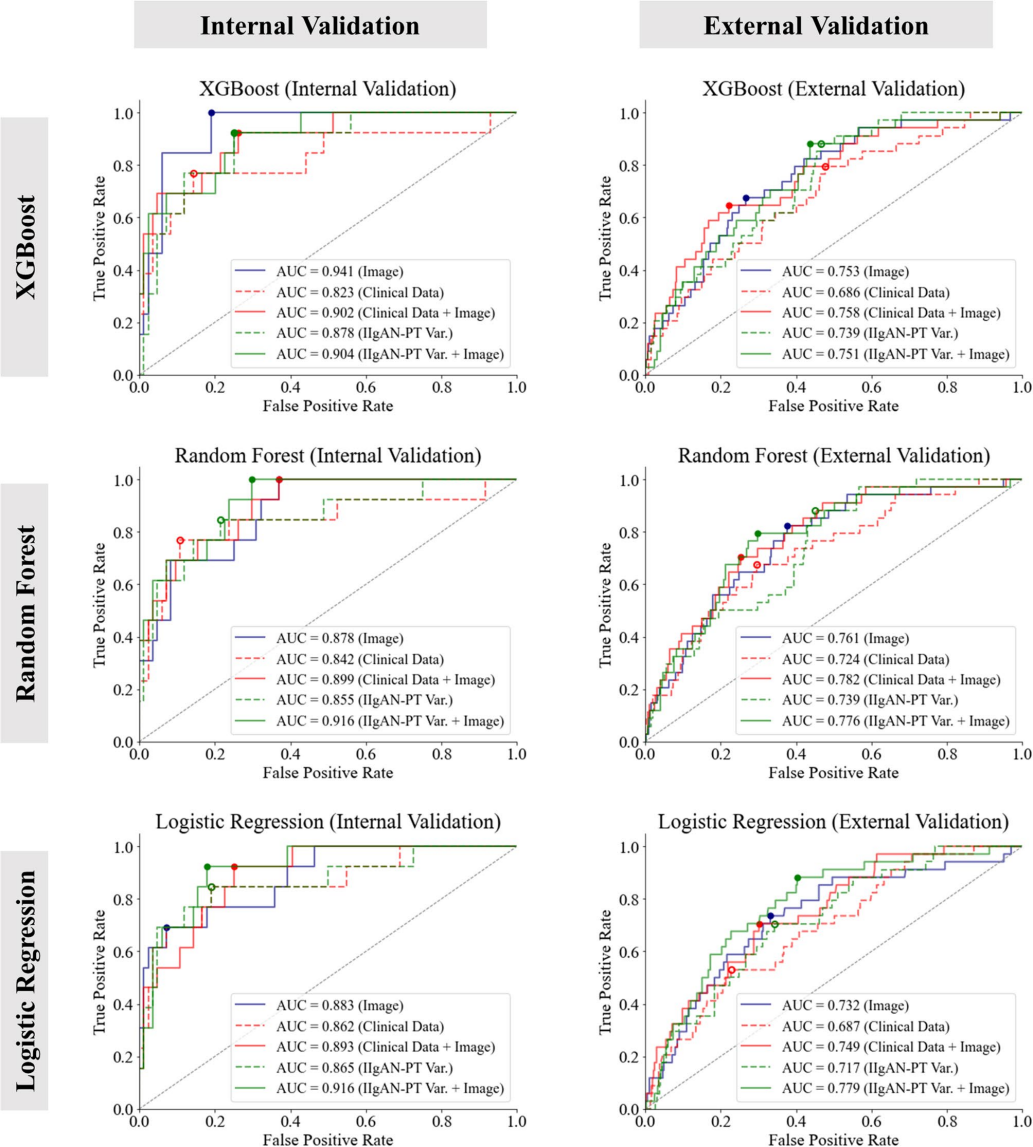
Receiver operating characteristic (ROC) curves of the kidney outcome prediction models, illustrating the performance of various classification models for predicting adverse kidney outcomes using image features, clinical data, or both, evaluated on internal and external validation sets.

The binary classification performance for predicting kidney outcomes was evaluated in terms of AUC (95% CIs). For internal validation, the AUC of the XGBoost model trained with the image features alone showed the highest value of 0.941 (0.851–1.000) among all models and conditions. Under other conditions in the XGBoost model, the AUC was 0.823 (0.680–0.967) when trained with basic clinical data and 0.902 (0.789–1.000) when image features were added. AUC was 0.878 (0.754–1.000) when trained with the clinical variables of IIgAN-PT and 0.904 (0.791–1.000) with image features added. In the random forest classifier models, AUC was 0.878 (0.754–1.000) with image features only. With basic clinical data alone, AUC was 0.842 (0.704–0.980), which increased to 0.899 (0.784–1.000) with image features added. With variables of IIgAN-PT, AUC was 0.855 (0.722–0.989), which increased to 0.916 (0.809–1.000) with image features added, achieving the highest AUC among the random forest models. Finally, with the logistic regression classifiers, the AUC of the model trained with image features alone was 0.883 (0.760–1.000). The model trained with the basic clinical data showed an AUC of 0.862 (0.731–0.993), which increased to 0.893 (0.775–1.000) with image features added. The model trained with the variables of IIgAN-PT showed an AUC of 0.865 (0.736–0.995), which increased to 0.916 (0.809–1.000) with image features added. Under the same conditions in the external dataset, the AUCs showed a similar trend, demonstrating higher AUC when the image features were added to basic clinical data or IIgAN-PT variables. The prediction performances of all models are summarized in Table 5, and the corresponding ROC curves are presented in Fig. 3 and Supplementary Fig. S1, illustrating the discriminative ability of each model across different input settings.

**Table 5:** Discriminative performances of the machine learning-based binary classification models for prediction of kidney outcomes.

Model	Input for training	Internal validation		External validation	
		97 (84:13)		399 (365:34)	
		AUC	*p*-value ^a^	AUC	*p*-value
XGB	Image features	0.941 (0.851–1.000)	–	0.753 (0.656–0.850)	–
	Clinical data	0.823 (0.680–0.967)	0.143 ^b^	0.686 (0.584–0.789)	0.167
	Image + clinical data	0.902 (0.789–1.000)	0.248 ^c^	0.758 (0.661–0.855)	0.088
	IIgAN-PT variables	0.878 (0.754–1.000)	0.194 ^b^	0.739 (0.640–0.837)	0.779
	Image + IIgAN-PT variables	0.904 (0.791–1.000)	0.551 ^d^	0.751 (0.653–0.848)	0.795
RF	Image features	0.878 (0.754–1.000)	–	0.761 (0.665–0.857)	–
	Clinical data	0.842 (0.704–0.980)	0.583	0.724 (0.624–0.824)	0.450
	Image + clinical data	0.899 (0.784–1.000)	0.353	0.782 (0.688–0.876)	0.190
	IIgAN-PT variables	0.855 (0.722–0.989)	0.675	0.739 (0.640–0.838)	0.652
	Image + IIgAN-PT variables	0.916 (0.809–1.000)	0.246	0.776 (0.681–0.871)	0.432
LR	Image features	0.883 (0.760–1.000)	–	0.732 (0.632–0.831)	–
	Clinical data	0.862 (0.731–0.993)	0.747	0.687 (0.585–0.789)	0.435
	Image + clinical data	0.893 (0.775–1.000)	0.512	0.749 (0.651–0.846)	0.149
	IIgAN-PT variables	0.865 (0.736–0.995)	0.783	0.717 (0.616–0.817)	0.791
	Image + IIgAN-PT variables	0.916 (0.809–1.000)	0.296	0.779 (0.685–0.873)	0.177

Values are presented as AUC (range of 95% confidence interval).

*AUC* area under the curve, *XGB* extreme gradient boosting classifier, *RF* random forest classifier, *LR* logistic regression classifier, *IIgAN-PT* international IgAN prediction tool.

^a^*p*-values, according to the Delong’s test, ^b^*p*-value versus image features, ^c^*p*-value versus clinical data, ^d^*p*-value versus IIgAN-PT variables.

The feature importance of each model was visualized in bar graphs, as presented in Supplementary Fig. S2, to determine the features that influenced the prognostic model the most. In models using the clinical data alone, the T score (tubular atrophy) and the patient’s age at biopsy were commonly ranked in the top 5 variables in the result. In the models that included image features, the notable variables were mostly the features associated with global sclerosis and no-lesioned glomeruli, which consistently ranked in the top 5 variables despite the inclusion of clinical data in training and development. Specifically, features related to the ratio and size of globally sclerotic and no-lesion glomeruli contributed additional prognostic information beyond clinical variables.

## Discussion

Accurate prognosis prediction in IgAN is essential for appropriate risk stratification and clinical management, necessitating the analysis of pathologic images^3^. In this study, deep learning and machine learning models were developed for automated morphometric analyses of glomeruli in biopsy specimens, which aimed to predict the prognosis of IgAN. This study presents several advantages. First, it provides a lesion-specific segmentation of glomeruli in PAS-stained WSIs, which are routinely used in clinical settings. Second, the model was trained and validated on a large, multi-institutional cohort, supporting its generalizability. Third, integration of deep learning and machine learning pipelines offers an objective method for quantifying glomerular morphology. The ability to extract quantitative, reproducible image features from routine WSIs reduces reliance on subjective scoring and enables automation of risk prediction. In addition, the proposed method may be applicable to other areas of pathology beyond kidney diseases. With appropriate annotation and task-specific adaptation, it could potentially be extended to cancer histopathology, enabling lesion-specific morphology analysis in tumor tissues.

A total of 1,241 WSIs of kidney biopsies were collected, and 17,124 annotated glomeruli were considered for developing and validating the prognostic model. The glomeruli were segmented and classified into five types of lesions using a deep learning model. The quantified features of the segmented glomeruli were used to predict the adverse kidney outcome of IgAN patients, using machine learning models. The prognostic performance of the image-based prognostic model was assessed by comparing it with clinical data-based models.

Several studies have previously attempted to contribute to predicting adverse kidney outcomes in IgAN patients by integrating digital pathology and deep learning methods. Some studies have developed glomerulus segmentation models for analyzing glomerulosclerosis and the overall assessment of kidney tissues in biopsy slides^15–17^. However, the models were not tested to validate their contribution to predicting kidney disease progression. Recently, deep learning-based Oxford classification methods have been proposed, such as the automated quantification of each element in the MEST-C score using Masson’s trichome-stained kidney WSIs and glomerular segmentation followed by classification of elements in Oxford classification^29,30^. However, PAS stain is classically recommended for Oxford score evaluation^31^, and some nephrologists claim the presence of possible prognostic information in kidney biopsy not covered by Oxford scores^32,33^. In another study, a deep learning predictive score for predicting kidney failure in IgAN by examining the entire WSI was proposed; however, it was a single cohort study, and external validation was absent^34^. A morphometric analysis has also emerged as a deep learning-based approach for quantitative kidney histopathology, allowing disease characterization, though its clinical application may be constrained by computational complexity and limited interpretability of high-dimensional features^35^. In this study, the deep learning and machine learning framework utilized the basic, interpretable morphologic features of various lesioned glomeruli using PAS-stained WSIs from multi-center IgAN cohorts, including seven medical institutions, to explore the prognostic ability. The glomerulus segmentation model showed acceptable performances in internal and external cohorts, and the segmented glomeruli feature-based prognostic classification models were similar to those developed using the clinical data.

The proposed multi-class glomerulus segmentation model accurately located the glomeruli and classified their lesion types. The model showed acceptably high performance in terms of the weighted average AP and DSC scores and comparable performances in internal and external cohorts, showing robustness across diverse environments. Furthermore, although the difference between the AUCs did not show statistical significance, the AUC of the prognosis prediction model increased with the addition of the image features in all three machine learning algorithms and internal and external cohorts. This increase in AUC with the addition of image features demonstrates that the image features contain meaningful information in addition to the clinical data. Particularly, mean arterial pressure, UPCR, and eGFR at biopsy were previously reported as the best recognized clinical predictors of kidney outcome^36^, and another study added tubulointerstitium (T parameter of the MEST-C) as a statistically predictive parameter for kidney failure^37^. Despite including these variables in the clinical data-based prediction models, the image feature-based models, or the addition of the image features, contributed to higher AUCs of the prognosis prediction models, establishing the comparability of the image features to clinical data.

The outcome prediction model was further evaluated through feature importance analysis. The T score of Oxford classification appeared as a common top-ranking feature in clinical data-based models^37^. However, when adding image features to the clinical data-based models, image features ranked higher than the clinical features. Moreover, the assessment of glomeruli with global sclerosis is important in the risk prediction of IgAN, although not encompassed by the Oxford classification. In this study, global sclerotic glomeruli within kidney WSIs were annotated and analyzed to improve the prognostic accuracy of IgAN, which likely influenced the model’s prediction performance. The image features associated with global sclerosis consistently ranked among the top 5 variables in all models with image features included. The ratio of non-lesional glomerular area also ranked highly, which suggests that a lower ratio, indicating a higher proportion of glomeruli with lesions, may reflect higher risk of adverse kidney outcomes. Interestingly, the urinary protein-to-creatinine ratio (UPCR), despite its known clinical relevance, showed relatively low importance in the clinical and image feature combined model (Supplementary Fig. S2). This may be due to the dominance of top-ranking image features, suggesting strong predictive value of the image features. Additionally, since feature importance rankings in tree-based models depend on hierarchical splits, they may not fully reflect multivariate interactions among correlated predictors.

**Fig. 4:**
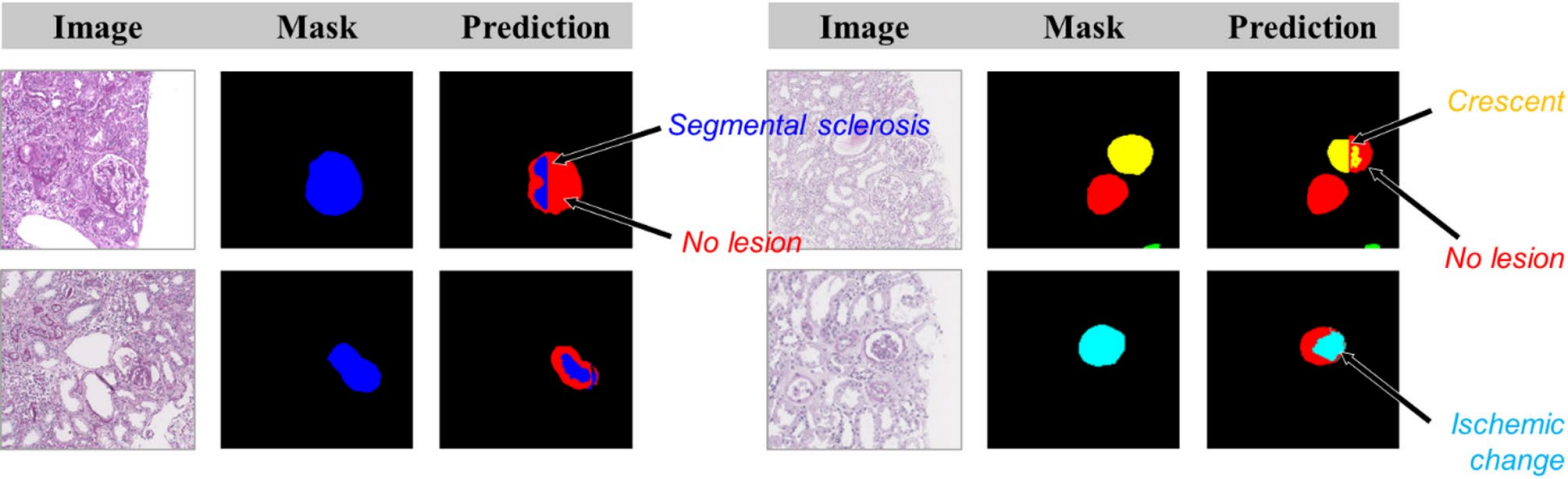
Examples of partially correct segmentations in glomeruli with segmental sclerosis, crescent, and ischemic changes. These cases show glomerular lesion areas that were only partially segmented, highlighting segmentation challenges.

While the glomerulus segmentation model exhibited acceptable performances across various cohorts, as indicated by the overall weighted average AP and DSC scores, relatively low performances were observed in glomeruli with crescents and ischemic changes. Such disparity in performance can be attributed to class imbalance. Deep learning methods are known to have inherent limitations when dealing with highly imbalanced datasets as bias persists toward the majority class^28^. In the training and development dataset, the number of glomeruli with no lesions was approximately 40 times that of glomeruli with crescents or ischemic changes. Although under-sampling and over-sampling were implemented before training to reduce this gap, deep learning models tend to overfit the over-sampled data, potentially limiting their generalizability across classes. The composite scarcity of instances in the training set and the similarities in morphological characteristics may further hinder effective training. Interestingly, most glomeruli with ischemic changes were misclassified as having no lesions. Furthermore, some segmental sclerosis, crescent, or ischemic changes exhibited only partially correct segmentations, with some regions predicted as having no lesion. Examples of these partial segmentations are shown in Fig. 4, where glomeruli with segmental sclerosis, crescent, or ischemic changes were only partially recognized by the model, with unaffected regions incorrectly predicted as no-lesion areas. These cases indicate the challenge in segmenting subtle lesions that coexist with normal glomerular structures. Particularly, when ischemic change is mild, a glomerulus with ischemic change may appear similar to a no-lesioned glomerulus. All these factors could reduce the DSC scores. Hence, although the DSC score is the standard evaluation metric for segmentation, this study also computed AP scores as an evaluation metric. The glomerulus annotations in this study aimed to approximate their locations and distinguish lesion types. Hence, each glomerulus was labeled with an accurate lesion type but may not always have precise boundaries. Although the AP score based on a 50% prediction overlap may overestimate segmentation performance, it evaluates whether predicted regions sufficiently cover the ground truth targets, whereas the DSC score is more sensitive to precise boundary agreement. Therefore, this study insists that reporting both AP and DSC metrics provides a more balanced evaluation of segmentation performance in this task.

It is important to address the limitations of this study. First, the dataset used for the segmentation model training was imbalanced, particularly for less common glomerular lesions such as crescents and ischemic changes. This imbalance may have restricted the model’s ability to more accurately segment rare lesion types. Second, the analysis was limited to glomerular features, without incorporating tubulointerstitial compartment or other image descriptors such as texture features. Since tubular atrophy and interstitial fibrosis are known to play a key role in kidney disease progression, and texture features are known as descriptors for characterizing glomerular structures, the absence of such data may limit the model’s predictive scope. Third, time-to-event analysis was not performed due to inconsistencies in follow-up durations across institutions. Incorporating survival analysis may provide additional insight in future studies by capturing temporal aspects of risk.

This study effectively analyzed the characteristics of various lesioned glomeruli from kidney biopsies using deep learning and machine learning methods to predict adverse kidney outcomes in IgAN patients. Despite the selected image features being morphological features based on simple and basic computer vision techniques, the image features in the proposed framework were comparable to the clinical data and may offer additional prognostic insights not covered by Oxford classification and IIgAN-PT variables. Moreover, its acceptable performance can assist clinicians with valuable information, and its practical application in real clinical settings is expected.

## Electronic supplementary material

Below is the link to the electronic supplementary material.

Supplementary Material 1

## Data Availability

The data used and/or analyzed during the current study are available from the corresponding authors upon reasonable request.
