# Supplementary material for: Deep learning-based quantitative analysis of glomerular morphology in IgA nephropathy whole slide images and its prognostic implications
Source: Sci Rep. 2025 Jul 2;15:23566. doi: 10.1038/s41598-025-09031-w (PMC12222963; doi:10.1038/s41598-025-09031-w)
Supplement: Supplementary file 1 — Supplementary Material 1 [file 41598_2025_9031_MOESM1_ESM.docx]

**Supplementary Table S1.** Total variables included in the clinical data-based prognostic model

| Input for Training | Variables |
| --- | --- |
| Clinical Data | Age, sex, renin-angiotensin-aldosterone system blockades, immunosuppressive drug, Oxford classification (M, E, S, T, C), diabetes mellitus, hypertension, body mass index, systolic BP, diastolic BP, hemoglobin |
| IIgAN-PT Variables | Age, renin-angiotensin-aldosterone system blockades, immunosuppressive drug, Oxford classification (M, E, S, T, C), mean arterial pressure, urine protein-to-creatinine ratio |

**Supplementary Table S2.** Total variables included in the image feature-based prognostic model

| Feature | Description |
| --- | --- |
| glom_num | Total number of glomeruli |
| glom_area | Total amount of area covered by glomeruli |
| *x*_area ^a)^ | Area of *x* glomeruli |
| *x*_area_ratio | Area of *x* glomeruli over total area of all glomeruli |
| *x*_hit | Number of *x* glomeruli |
| *x*_hit_ratio | Number of *x* glomeruli over total number of all glomeruli |
| *x*_mjax | Average length of major axis of *x* glomeruli |
| *x*_mjax_std | Standard deviation of length of major axis of *x* glomeruli |
| *x*_mnax | Average length of minor axis of *x* glomeruli |
| *x*_mnax_std | Standard deviation of length of minor axis of *x* glomeruli |
| *x*_compact | Average compactness of *x* glomeruli |
| *x*_compact_std | Standard deviation of compactness of *x* glomeruli |
| *x*_eccent | Average eccentricity of *x* glomeruli |
| *x*_eccent_std | Standard deviation of eccentricity *x* glomeruli |
| *x*_solid | Average solidity of *x* glomeruli |
| *x*_solid_std | Standard deviation of solidity of *x* glomeruli |
| *x*_round | Average roundness of *x* glomeruli |
| *x*_round_std | Standard deviation of roundness of *x* glomeruli |

^a)^ *x* exists for *nl* (no lesion), *gs* (global sclerosis), *ss* (segmental sclerosis), *cr* (crescent), *isc* (ischemic change)


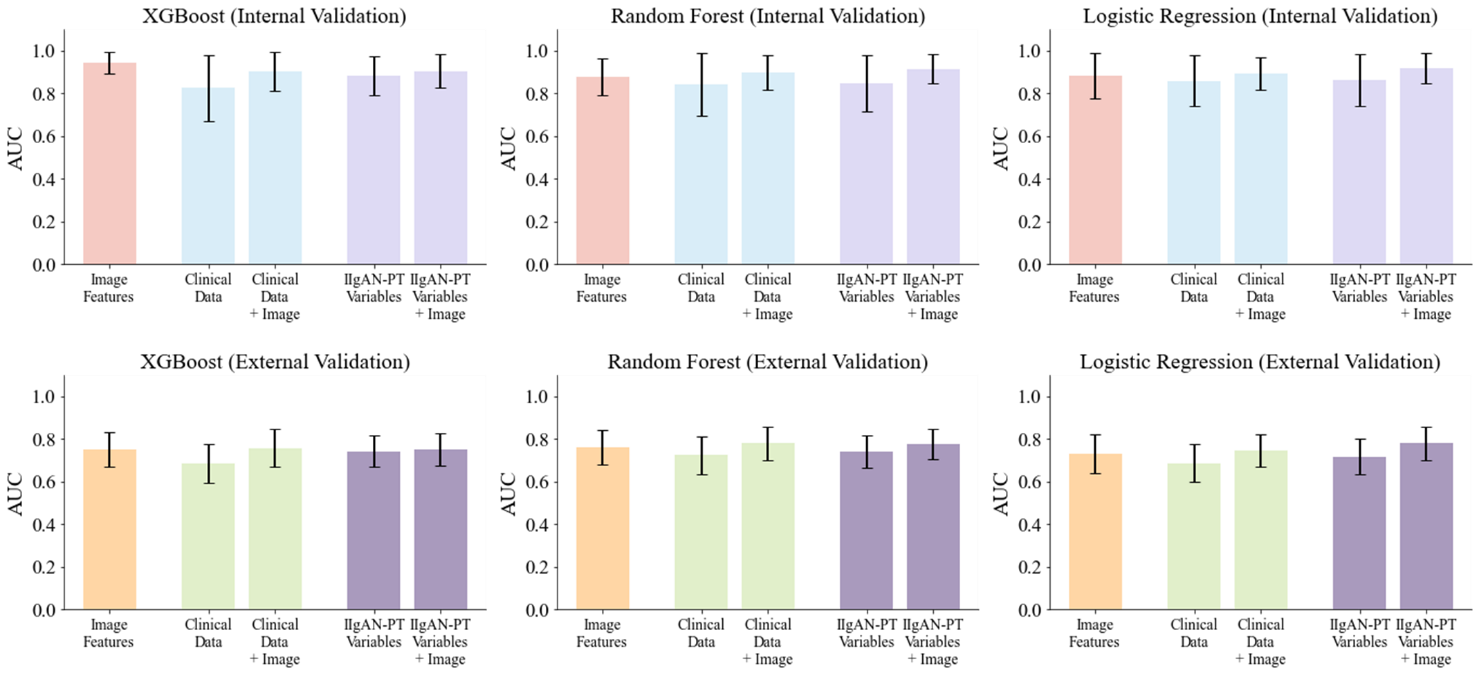
 **Supplementary Figure S1.** Visualization of the performance of machine learning-based binary classification models for prediction of kidney outcomes.


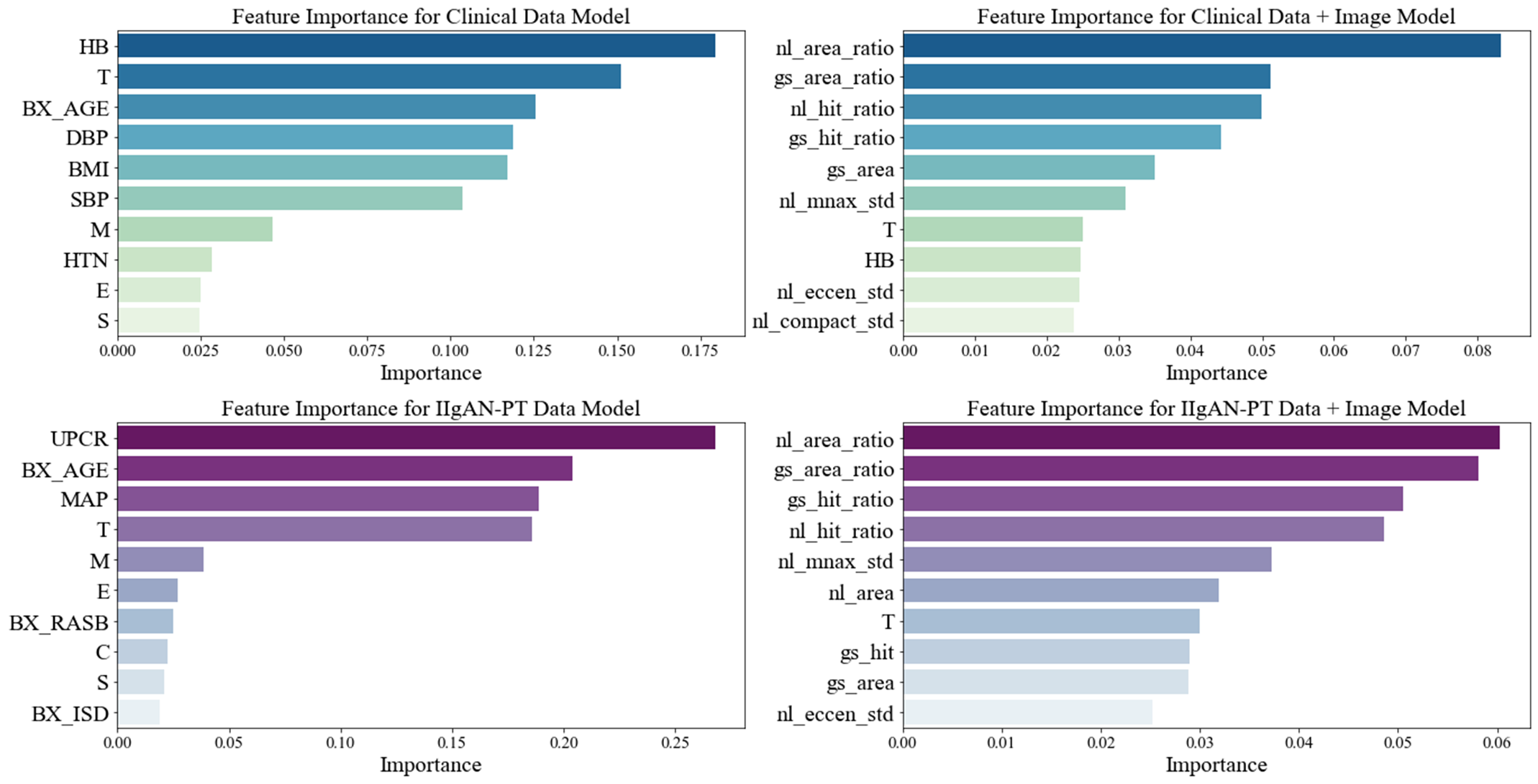


**Supplementary Figure S2.** Feature importance plots for the kidney outcome prediction models
